# Comparison of Folate Receptor Targeted Optical Contrast Agents for Intraoperative Molecular Imaging

**DOI:** 10.1155/2015/469047

**Published:** 2015-09-28

**Authors:** Elizabeth De Jesus, Jane J. Keating, Sumith A. Kularatne, Jack Jiang, Ryan Judy, Jarrod Predina, Shuming Nie, Philip Low, Sunil Singhal

**Affiliations:** ^1^Division of Thoracic Surgery, Department of Surgery, University of Pennsylvania and Philadelphia VA Medical Center, Philadelphia, PA 19104, USA; ^2^On Target Laboratories, Inc., West Lafayette, IN 47906, USA; ^3^Departments of Biomedical Engineering and Chemistry, Emory University, Atlanta, GA 30322, USA; ^4^Department of Chemistry, Purdue University, West Lafayette, IN 47907, USA

## Abstract

*Background*. Intraoperative imaging can identify cancer cells in order to improve resection; thus fluorescent contrast agents have emerged. Our objective was to do a preclinical comparison of two fluorescent dyes, EC17 and OTL38, which both target folate receptor but have different fluorochromes. *Materials*. HeLa and KB cells lines were used for *in vitro* and *in vivo* comparisons of EC17 and OTL38 brightness, sensitivity, pharmacokinetics, and biodistribution. *In vivo* experiments were then performed in mice.* Results*. The peak excitation and emission wavelengths of EC17 and OTL38 were 470/520 nm and 774/794 nm, respectively. *In vitro*, OTL38 required increased incubation time compared to EC17 for maximum fluorescence; however, peak signal-to-background ratio (SBR) was 1.4-fold higher compared to EC17 within 60 minutes (*p* < 0.001). Additionally, the SBR for detecting smaller quantity of cells was improved with OTL38. *In vivo*, the mean improvement in SBR of tumors visualized using OTL38 compared to EC17 was 3.3 fold (range 1.48–5.43). Neither dye caused noticeable toxicity in animal studies. *Conclusions*. In preclinical testing, OTL38 appears to have superior sensitivity and brightness compared to EC17. This coincides with the accepted belief that near infrared (NIR) dyes tend to have less autofluorescence and scattering issues than visible wavelength fluorochromes.

## 1. Introduction

Complete surgical resection of malignant tissue is the single most effective method for managing a patient with a solid tumor [1]. However, failure to obtain complete disease clearance due to an incomplete resection such as positive tumor margins or metastatic cancer cells in lymph nodes is a major challenge and can occur in 20–60% of cancer operations [1]. Intraoperative molecular imaging has emerged as an innovative approach to overcome this problem [2–4]. Tools such as fluorescence guided imaging surgery, which utilize fluorescent probes and sensitive optical imaging devices, provide real-time information to surgeons about potentially malignant tissue to improve disease clearance.

Recently, two contrast agents, EC17 and OTL38, have been proposed to image ovarian and lung adenocarcinomas during surgery [5, 6]. These agents are similar in that they target the folate receptor alpha (FR*α*) via a folate ligand. FR*α* is a useful target for intraoperative molecular imaging of ovarian and lung adenocarcinomas. Folate, a B vitamin (molecular weight 440), plays a key role in metabolic processes involved in DNA and RNA synthesis, epigenetic processes, cellular proliferation, and survival of lung adenocarcinomas [7]. There are 4 members of the folate receptor family, though only FR*α* and FR*β* bind folate with high affinity. FR*α* is naturally expressed at the luminal surface of polarized epithelial cells; thus these cells do not bind serum folate [7–10]. On the other hand, lung adenocarcinomas express FR*α* (1–3 million receptors/cancer cell) and bind serum folate 10^3^–10^4^ times more avidly than normal pulmonary epithelial cells [11–13]. Thus, FR*α* provides a reasonable molecular target on pulmonary adenocarcinomas for diagnostic purposes.

Although EC17 and OTL38 have the same ligand, they have two different fluorochromes: EC17 contains a fluorescein dye and OTL38 contains a cyanine dye. Fluorescein is in the visible wavelength and the cyanine is in the NIR range. There are biological advantages to NIR imaging due to the decreased autofluorescence and less rejected scattering that occurs with visible fluorophores. However, fluorescein has been well tested for several decades and has a low toxicity profile, whereas other NIR dyes (except for indocyanine green) are relatively untested in humans. For these reasons, the goal of this study was to generate preclinical data to compare two optical contrast agents, EC17 and OTL38, both of which target the same receptor, FR*α*.

## 2. Materials and Methods

### 2.1. Cell Lines

The murine lung cancer cell line, TC1, was derived from primary lung epithelial cells from C57BL/6 mice and transformed with the c-Ha-ras oncogene [14, 15]. HeLa is the oldest and most commonly used human cancer cell line. It was established from human cervical papillomavirus (HPV 18) related carcinoma. KB is a human carcinoma cell line originally believed to be of oropharyngeal origin but was eventually found to be a derivative of HeLa [16]. TC1, KB, and HeLa cell lines were cultured, maintained, and passaged in RPMI (RPMI 1640 folate deficient Medium, Gibco Life Technologies) supplemented with 10% fetal bovine serum (FBS; Hyclone), 1% penicillin/1% streptomycin, and 1% glutamine. Cell lines were regularly tested and maintained negative for* Mycoplasma* and were maintained in 5% CO_2_ at 37°C, in a humidified incubator.

### 2.2. Mice

Female C57BL/6 mice were purchased from Jackson Laboratories and female NOD.Cg-*Prkdc* ^*scid*^ *Il2rg* ^*tm1Wjl*^/SzJ mice were bred at the CHOP Barrier at the Colket Translational Research Building at the Children's Hospital of Philadelphia. The mice were maintained in conditions approved by the Animal Care and Use Committees of the Children's Hospital of Philadelphia and the University of Pennsylvania and in agreement with the Guide for the Care and Use of Laboratory Animals.

### 2.3. Reagents

EC17 was synthesized by a folate (vitamin B9) and fluorescein isothiocyanate (FITC) conjugated through an ethylenediamine spacer to produce folate-FITC, with a molecular weight of 917 kDa. FITC is a derivative of fluorescein functionalized with an isothiocyanate reactive group. The folate-FITC conjugate forms a negatively charged fluorescent molecule that specifically targets cell-surface FR*α* and is subsequently internalized into the cytoplasm [12, 17]. OTL38 was synthesized by a folate ligand as well and a cyanine backbone dye with a molecular weight of 1414.42 kDa. All vials of EC17 and OTL38 were supplied by On Target Laboratories, Inc. (West Lafayette, IN).

### 2.4. Near-Infrared and Fluorescence Imaging Platforms

The GloMax Multi Detection System (Promega, Madison, WI) was used in fluorimeter operation mode to quantify EC17 and OTL38 fluorescence from samples placed into 96-well microplates. Wavelength matched LEDs provide the excitation light. A PiN-photodiode top-reads the amount of emission. The SpectraMax M5 Multi-Mode Microplate Reader (Molecular Devices, Sunnyvale, CA) was used to quantify NIR fluorescence. This fluorimeter uses a 50-watt xenon light source and has a wavelength range from 250 to 850 nm. A photomultiplier top-reads the emission intensity.

The “Flocam” is a home built digital imaging system based on a dual CCD camera system previously described [18] (BioVision Technologies Inc., Exeter, PA). The system uses two QIClick digital CCD cameras from QImaging (British Columbia, Canada), one for white brightfield and one for fluorescence overlay. The cameras have 696 × 520 pixel resolution and have a fluorescence exposure time of 20–200 ms. Each camera runs on 6 W supplied through a Firewire interface. The light source is a Spectra × Light Engine (Lumencor, Inc., Beaverton, OR). Six special-order NIR bandpass filters are employed to produce the excitation light. Using ImageJ, ROI measurements of the tumor and normal muscle were quantified and a signal-to-background ratio (SBR) was calculated. Positive and negative controls were used for all images.

### 2.5. *In Vitro* Models

KB, HeLa, and TC1 cells were plated on a cell culture treated 6-well plate (Corning Costar cell culture plates) and incubated for 16 hours. Once confluent, EC17 was added to one plate of cells, while OTL38 was added to another. The cells were incubated and sealed in a light-protected environment for 45 minutes. Cells were then washed 3 times with PBS and plated and underwent fluorescence microscopy.

### 2.6. Murine Flank Tumor Model

All mice were upheld in pathogen-free environments that were maintained on a 12-hour light/dark cycle with normal access to food and water. Experiments were conducted at 8 weeks of age or older. All experimental procedures were maintained and were in compliance with protocols approved by the Animal Care and Use Committee at the University of Pennsylvania and the Children's Hospital of Philadelphia. A total of 50 NOD/scid mice were used in order to test both EC17 and OTL38. Mice were injected subcutaneously in the flank with 1.2 × 10^6^ TC1 cells (C57BL/6 mice), 1.0 × 10^6^ HeLa cells (NOD.Cg-*Prkdc* ^*scid*^ *Il2rg* ^*tm1Wjl*^/SzJ mice), or 1.0 × 10^6^ KB cells (NOD.Cg-*Prkdc* ^*scid*^ *Il2rg* ^*tm1Wjl*^/SzJ mice). Tumor cells for subcutaneous flank injections were suspended in 100 *μ*L of PBS and injected under an IACUC approved protocol. Tumor volume was calculated using the formula (3.14 × long-axis × short-axis^2^)/6. Once tumor volume reached approximately 300 mm^3^ half of the mice were injected with 0.1 mg/kg of EC17 and the other half with 0.1 mg/kg of OTL38 via tail vein. Three hours later, the fluorescence of tumors was measured using Flocam. Of note, mice were fed an exclusively folate deficient chow (Harlan Laboratories) on the day of inoculation until endpoint.

### 2.7. Biodistribution Studies

5 mice with flank tumors (KB and HeLa) (total *N* = 5) were given 0.01 mg/kg of EC17 or OTL38 via tail vein injection. Twenty-four hours later, mice were euthanized by inhalation of CO_2_ followed by cervical dislocation. In order to assess the distribution of each reagent, the tumor, heart, lung, stomach, liver, spleen, pancreas, small bowel, large bowel, kidneys, bone, fat, and muscle were harvested and then imaged in *λ*_520_ or *λ*_820_.

### 2.8. Immunohistochemical Staining

Tissue specimens were harvested and bisected with one-half placed either in Tissue-Tek OCT and stored at −80°C or in formalin for paraffin sectioning. Frozen tumor sections were prepared as previously described [19]. To detect FR*α*, the monoclonal mouse antibody Mab343 (Morphotek Inc., PA) was used.

### 2.9. Fluorescence Microscopy

Fluorescence microscopy was performed using an Olympus IX51 fluorescent microscope equipped with a fluorescein specific filter set (Chroma 49012). Fluorescence microscopy for OTL38 was performed using an Olympus IX81 motorized inverted microscope equipped with an excitation filter of 780 nm and an emission wavelength of 830 nm (Chroma 49030). Image capture was achieved using a PixeLink NIR CCD camera (PL-B741EU). Background readings were taken from adjacent muscle and subcutaneous tissue in order to generate a signal-to-background ratio (SBR). All readings were done in quadruplicate.

### 2.10. Data Analysis

In order to quantitate the tissue fluorescence, we used region-of-interest software and HeatMap plugin within ImageJ (http://rsb.info.nih.gov/ij/; public domain free software developed by National Institutes of Health). A background reading was taken from adjacent normal muscle tissue in mice (e.g., gluteal muscle) in order to generate a background value (SBR). For experiments comparing differences between 2 groups, unpaired Student's *t*-tests were used. For studies comparing more than 2 groups, ANOVA was implemented. Differences were considered significant when *P* < 0.05. Data are presented as mean (SE), unless otherwise noted. For purposes of consistency, we set data acquisition times to 30 milliseconds on all imaging devices.

## 3. Results

### 3.1. Evaluation of Optical Properties of Folate-Targeted Fluorophores

**Figure 1:**
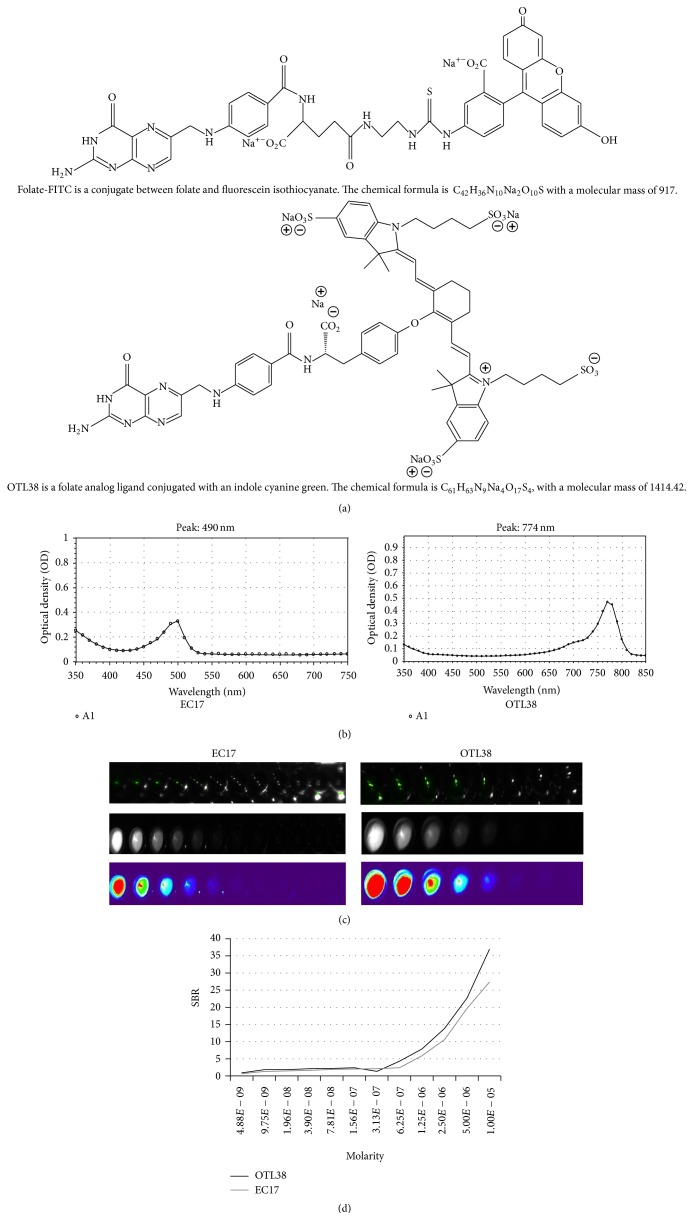
(a) The chemical structure of EC17 and OTL38; (b) biophysical properties of EC17 and OTL38 including excitation spectra; EC17 peak *λ*_ex_ 494 nm and OTL38 peak *λ*_ex_ 774 nm; (c) light intensity measured with Flocam; and (d) the luminometer of serial dilutions of EC17 and OTL38.

In order to confirm the excitation and emission spectra of EC17 and OTL38 (Figure 1(a)), 10 nM aliquots were measured with a luminometer. The peak excitation (*λ*_ex_) and emission (*λ*_em_) wavelengths of EC17 and OTL38 were 470/520 nm (Stokes shift 50 nm) and 774/794 nm (Stokes shift, 20 nm), respectively (Figure 1(b)). EC17 had superior Stokes shift that can allow for better discrimination and less overlap in developing a camera filtering system. Next, seven serial dilutions of 200 *μ*L aliquots of EC17 and OTL38 (ranging from 0 M to 1.0 × 10^−6^ M) were imaged first with Flocam (Figure 1(c)) and then measured in a luminometer in order to detect signal intensity (Figure 1(d)). Sterile water was used for background measurements. Signal-to-background ratios (SBR) were calculated for each sample by dividing the luminometer intensity of each reagent concentration divided by the intensity of sterile water. We found that the SBR of both reagents increased as the concentration was increased on a linear logarithmic scale. The SBR of EC17 ranged from 0.63 to 27.4, while the SBR of OTL38 was significantly higher and ranged from 0.92 to 37.0.

### 3.2. *In Vitro* Signal-to-Background Measurements

To confirm our imaging systems were calibrated and capable of identifying fluorescence, we placed a 25 *μ*L test drop of 1 *μ*M EC17 and OTL38 on a parafilm laboratory plastic film and recorded fluorescence over various integration times 10 ms, 30 ms, and 100 ms. The fluorescence was readily detectable at each time point with clear borders (data not shown).

**Figure 2:**
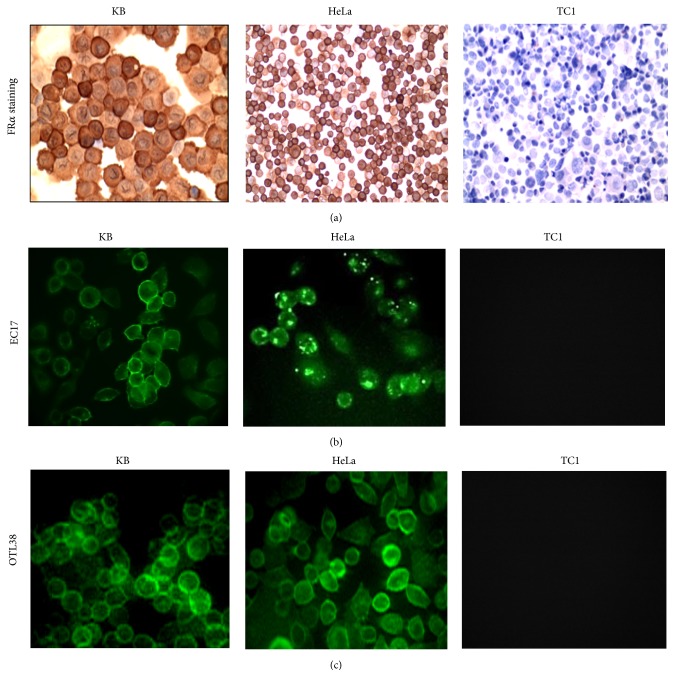
KB, HeLa, and TC1 cells folate receptor *α* staining (a) followed by KB, HeLa, and TC1 cells cocultured with fluorescent probes and imaged at 200x using a confocal microscope (b) and (c). Fluorescence was demonstrated in the viable cancer cells. (a) FR*α* staining (from left to right) of KB cells positive for folate receptor *α*. HeLa FR*α* staining also positive for folate receptors. TC1 cells staining negative for FR*α*. (b) Fluorescent imaging (bottom left) highlights KB cells fluorescing with EC17 fluorescence dye at 2.50 *μ*M, followed by fluorescent imaging of HeLa cells with EC17. Imaging (top right) exhibits negative coculture for TC1. (c) Near-infrared fluorescent imaging (bottom left) displays KB cells fluorescing with OTL38 fluorescence dye at 2.50 *μ*M. The following image demonstrates HeLa cells fluorescing with EC17. TC1 negative for OTL38 fluorescence.

Next, in order to determine which agent had the highest signal-to-background ratio (SBR), several lung cancer cell lines (KB, HeLa, and TC1) were first identified. Cytospins were prepared and immunostained for FR*α*. Staining demonstrated strong (3+) positivity of KB and HeLa and no FR*α* expression in TC1 (Figure 2(a)); thus TC1 was chosen as a negative control.

Then, 1*e* ^6^ KB cells were incubated with 0.1, 1.0, 2.5, 5.0, or 10.0 *μ*M of EC17 and OTL38. Cells were cocultured for 0, 5, 15, 30, 60, 120, and 240 minutes and then rinsed with PBS and imaged (Figures 2(b) and 2(c)). Background fluorescence was measured using cells that had not been incubated with the fluorophores. Also, background readings were taken from TC1 cells (e.g., FR*α* negative). There was negligible autofluorescence from the TC1 background readings. The mean background readings for TC1 were 231 ± 57.

**Figure 3:**
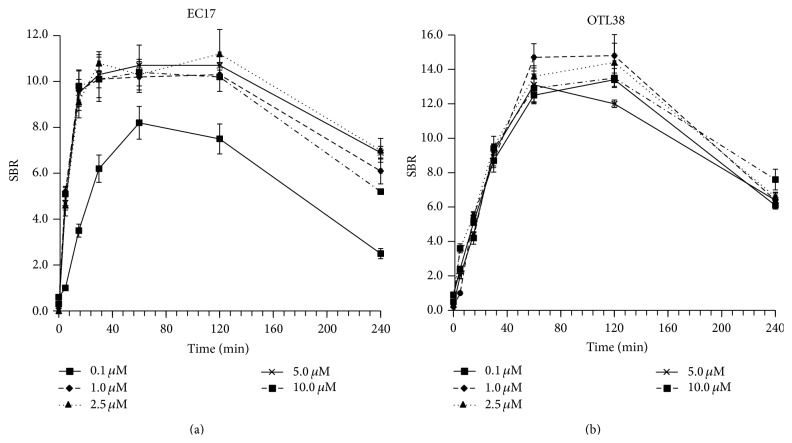
Time kinetics and dose titration of EC17 and OTL38 in representative KB cell line showing peak fluorescence* in vitro* at 30 minutes to 2 hours. OTL38 had a higher signal-to-background ratio compared to EC17 by 60 minutes.

We found that the SBR for EC17 was less than 5.2 (range 0.2–5.2) for the first 5 minutes after coculturing. However, by 30 minutes, the SBR was >10.6 (mean 11.0; range 10.6–11.8) for all dilutions except 0.1 *μ*M. The SBR was not significantly different (*P* > 0.2) from 30 minutes to 120 minutes; however it was markedly reduced by 240 minutes (Figure 3(a)).

Similar experiments were then repeated with OTL38. Again, we found the SBR for OTL38 was less than 5.5 (range 0.2–5.5) for the first 15 minutes after coculturing. After 60 minutes, the SBR was above 12.0 (mean 14.9; range 12.0–16.8) (Figure 3(b)). No dilution had any significant difference (*P* > 0.3) after 30 minutes at all designated concentrations.

Thus, we concluded that although OTL38 required increased incubation time for maximum fluorescence, its peak SBR was markedly increased (mean increase 1.4-fold) compared to EC17 by 60 minutes (*P* < 0.001).

### 3.3. *In Vitro* Sensitivity

**Figure 4:**
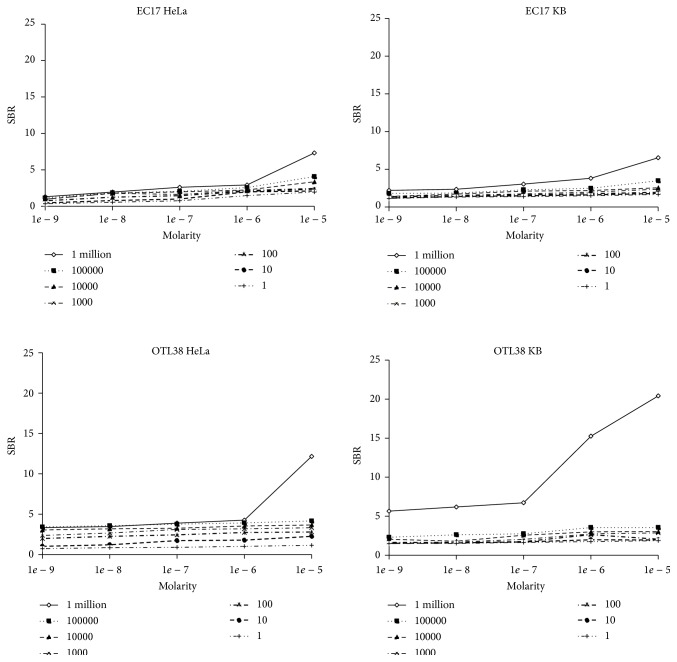
HeLa and KB cells were cultured with EC17 and OTL38 to measure sensitivity for small quantities of tumor cells. For both cell lines, OTL38 had a higher SBR than EC17, particularly at the smaller quantities of cells and with lower dilutions of the fluorochromes.

During intraoperative imaging in humans, the goal is to identify the smallest quantity of disease. In practical intraoperative applications, this translates to the highest SBR for the least number of tumor cells. In order to compare the sensitivity of EC17 and OTL38 for tumor cells, we seeded 6 flat-bottom well plates (950 mm^2^) with either KB or HeLa cells. The cells were allowed to adhere for 24 hours, and then they were cocultured with EC17 and OTL38 at either 0.1, 1.0, 2.5, 5.0, or 10.0 *μ*M. Cells were washed with PBS and then imaged after 45 minutes (Figure 4).

For HeLa cancer cells, OTL38 was more sensitive than EC17. The SBR of EC17 for HeLa cells ranged from 0.97 to 7.32 depending on the molarity and concentration of cancer cells. For OTL38, the SBR ranged from 0.90 to 12.16. The range of improved SBR of OTL38 compared to EC17 was from 1.07 to 3.15, with a mean improvement in SBR from EC17 to OTL38 being 1.54-fold (*P* < 0.002). For KB cancer cells, OTL38 was also more sensitive than EC17. The SBR of OTL38 for KB cells ranged from 1.13 to 6.53, whereas, for OTL38, the SBR ranged from 1.01 to 12.16. The range of improved SBR of OTL38 for KB cells compared to EC17 ranged from 1.10 to 4.02, with a mean improvement in SBR of 1.47-fold (*P* < 0.004).

Importantly, for both HeLa and KB models, SBR was significantly better with OTL38 when there was lower concentration of cancer cells. For example, for HeLa, the SBR for 0.01 cells/mm^2^ ranged from 1.10 to 2.54 for EC17, and the SBR for 0.01 cells/mm^2^ ranged from 1.81 to 3.48 for OTL38. Similarly for KB, the SBR for 0.1 cells/m^2^ ranged from 1.42 to 2.55 for EC17, and the SBR for 0.1 cells/mm^2^ ranged from 2.37 to 3.67. Thus, the SBR for detecting smaller quantity of cells was significantly improved with OTL38, and this effect was less pronounced (though present) at larger quantities of cancer cells.

### 3.4. *In Vivo* Optical Imaging

**Figure 5:**
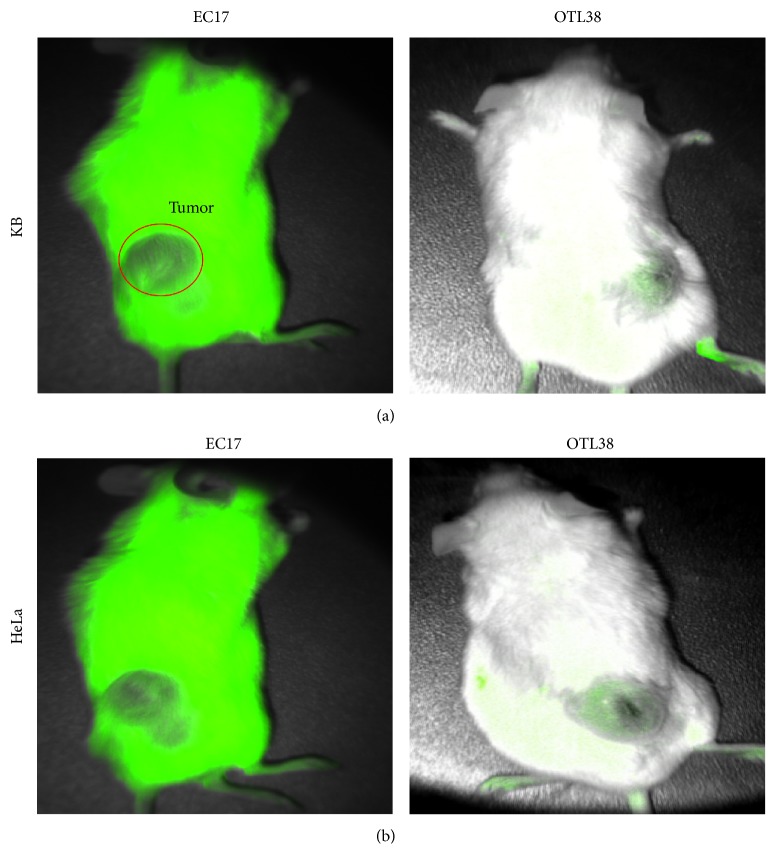
Comparison of* in vivo* tumor imaging in murine model. (a) OTL38 fluorescence imaging of mouse followed by EC17 fluorescence imaging, both with KB tumor burden. (b) OTL38 fluorescence imaging of mouse followed by EC17 fluorescence imaging, both with HeLa tumor burden.

In order to determine which fluorophore would be superior for* in vivo* imaging, we tested each contrast agent on a small animal model. We used 1*e* ^6^ KB and HeLa cells in NOD/scid mice. Animal flanks were subcutaneously injected with tumor cells, and then they were monitored until they reached 250 mm^3^. Once they reached the designated volume, the animals were injected with 0.01 mg/kg EC17 or OTL38 via tail vein (Figure 5). We found the mean fluorescence signal from the animals injected with EC17 to be 42,234 ± 12,234 au and the fluorescence signal from the OTL38 to be 58,234 ± 8,324. The background signal from the gluteus muscle in both cohorts with EC17 and OTL38 was negligible (mean < 8,324). However, EC17 had significant technical issues that required image processing because of the natural autofluorescence from the white fur of NOD/scid mice. Once this issue was accounted for, the mean improvement in SBR of tumors visualized using OTL38 compared to EC17 was 3.3-fold (range 1.48–5.43).

**Figure 6:**
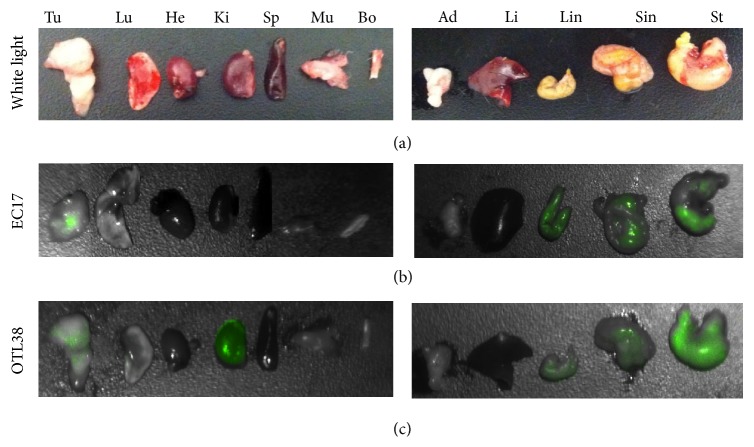
Biodistribution for clearance of tracer from *N* = 2 animals. Representative brightfield (a), fluorescent OTL38 (b), and fluorescent EC17 (c) imaging of resected vital tissue excised 2 hours after intravenous (i.v) injection. Tu, tumor; Lu, lungs; He, heart; Ki, kidneys; Sp, spleen; Mu, muscle; Bo, bone; Ad, adipose; Li, liver; Sin, small intestine; Lin, large intestine; St, stomach.

We next studied the dye distribution and the systemic toxicity of EC17 and OTL38 in mice. Animals with flank tumors were euthanized 6 hours after EC17 and OTL38, and the internal organs were harvested for fluorescence imaging. As shown in Figure 6, the tumor and peritumoral tissues, including but not limited to a lobe of the lung, heart, kidney, spleen, liver, bone, fat, and muscle, were isolated, harvested, and imaged. The majority of the EC17 and OTL38 accumulated in the digestive system, mostly localized in the stomach, small intestines, and large intestines as shown. There was significant fluorescence in the flank tumors of both mice. No signal was found in the lung, heart, spleen, muscle, bone, fat, or liver. The OTL38 was fluorescent in the kidneys, whereas the EC17 was not. All animals survived all studies. There were no signs of acute toxicity in any of the animals.

## 4. Discussion

Molecular imaging of ovarian and lung adenocarcinomas has emerged as a new technology to identify tumors during surgery. Two contrast agents, EC17 and OTL38, are being developed to image these tumors types in humans. Both agents bind the same target (e.g., FR*α*) via the folate ligand; however, their fluorochromes are vastly different. In preliminary studies from the producers of these tracers, On Target Laboratories, Inc. (West Lafayette, IN), OTL38 seems to have a higher binding affinity for folate receptor; however ongoing tests are currently comparing the pharmacokinetic and photophysical profiles of EC17 and OTL38. EC17 contains the fluorescein fluorochrome and has a spectral wavelength of 490–530 nm, whereas OTL38 has a cyanine dye backbone that emits in the region of 774–794 nm. Our goal was to present some comparative aspects of these dyes in preparation for clinical use. Based on our data, we postulate that OTL38 will have superior clinical efficacy.

Fluorophores in the fluorescein spectral range have decreased tissue penetration and increased autofluorescence. Infrared dyes, however, have deeper tissue penetration, a significant reduction in scattering, and less autofluorescence. Infrared dyes have optimal imaging properties due to the fact that wavelength is not in the normal bioluminescence of surrounding tissue. Collagen and elastin are known to fluoresce in the ultraviolet range (10 nm–400 nm) and visible spectrum (400–700 nm) [20]. Therefore, there is a window of opportunity from 700 to 900 nm where tumor fluorescence can be detected* in vivo* with decreased background signal and increased tissue penetration. Our murine data confirms these findings.

We also found that OTL38 had superior fluorescence* ex vivo* when background signal was controlled. We used similar models; thus the receptor frequency was controlled. This suggests that OTL38 has superior brightness and/or our imaging system was more sensitive to NIR signals. We used luminometry in an attempt to overcome bias in our device design, and still OTL38 showed superior signal emission in this setting as well. We did find that the OTL38 incubation time was slightly longer* in vitro*. However, we do not believe this has any significant practical implications because the infusion into the patient would occur prior to surgery. Thus, short time interval differences will unlikely affect the natural flow of an operation.

These results are promising; however, the translation of preclinical murine data to humans has been controversial [21, 22]. In our experiences, we have performed four clinical trials in intraoperative imaging using indocyanine green, which is a nontargeted agent; however we have been disappointed with the correlation of murine data to human data. Specifically, tumor fluorescence of murine models is more reliable than we have found in our human data. This inconsistency may be due in large part to the lack of targeted specific tracer binding. Additionally, we also believe the challenge lies with the differences in pharmacokinetics, liver metabolism, and body compartment sizes. Although murine models are mammalian in origin, the tumors tend to be relatively heterogeneous. Human tumors are more chronic and inflammatory in nature; thus they tend to have more genetic variability. Based on our experiences with the variance of success between murine and human imaging, we may need to consider both EC17 and OTL38 for human trial, even though the data is strongly in favor of OTL38.
